# Heat Transfer Performance of Circular Ultra-Thin Vapor Chamber with Superhydrophilic Patterned Microstructure for LED Lamp Cooling

**DOI:** 10.3390/mi17101202

**Published:** 2026-10-17

**Authors:** Tengqing Liu, Yaokang Zhang, Yaoxun Feng, Shuangfeng Wang, Qianxi Zhang

**Affiliations:** 1 College of Ocean Engineering and Energy, Guangdong Ocean University, Zhanjiang 524088, China; tqliu1990@hotmail.com (T.L.); fengyaoxun@126.com (Y.F.); zhangqx@gdou.edu.cn (Q.Z.); 2 Key Laboratory of Heat and Mass Transfer and Low-Carbon Conversion of the Ministry of Education, School of Chemistry and Chemical Engineering, South China University of Technology, Guangzhou 510640, China; sfwang@scut.edu.cn

**Keywords:** LED lamp cooling, ultra-thin vapor chamber, superhydrophilic patterned microstructure, condensation temperature, heat transfer performance

## Abstract

This paper presents a circular ultra-thin vapor chamber (UTVC) with a thickness of 1.3 mm for LED lamp thermal management. Superhydrophilic patterned microstructure (SPMS) arrays are fabricated on the upper and bottom covers to replace the traditional wick structure. The heat transfer performance of the ultra-thin vapor chamber was experimentally investigated under different cooling water temperatures in the horizontal orientation. The cooling water temperatures range from 25 to 40 °C, and the input-heat loads range from 5 to 25 W. The results show that for the proposed UTVC with SPMS arrays, the temperatures rise rapidly with increasing input-heat load and then level off after a certain period, with the temperature rise being considerably smaller than that of the ceramic heater. The condenser temperature difference increases with both the input-heat load and the cooling water temperature, and it ranges from 1.2 to 7.3 °C. Both the heater-to-vapor-chamber thermal resistance and the system thermal resistance increase with a higher input-heat load and a lower cooling water temperature. The heater-to-vapor-chamber thermal resistance ranges from 0.80 to 1.34 K/W, and the system thermal resistance ranges from 3.10 to 4.13 K/W.

## 1. Introduction

Light-emitting diodes (LEDs) are widely used because of their low cost, long lifespan, and high light efficiency in many lighting applications, such as flashlights, automotive headlamps, street lamps, and television backlights [1,2]. Because only 20% of the input energy for LED lamps is converted into optical power, the rest is dissipated as heat [3]. LED chips are miniaturized, and the operating space is limited, but their heat flux is high. Heat accumulation raises the LED chip temperature, which reduces electro-optical conversion efficiency, shifts the light wavelength, and degrades the phosphor lifespan [4]. Therefore, novel components that can effectively dissipate heat in limited space have been developed to address this issue.

Vapor chambers (VCs), as two-dimensional two-phase heat-transfer components, are one of the most effective and compact technologies for LED lamp cooling due to their low thermal resistance and good temperature uniformity. They utilize the latent heat of phase change of the working fluid to rapidly spread heat from a hot spot to a large condenser area. Therefore, researchers have paid attention to vapor chambers for thermal management of LED lamps. Huang et al. [5] developed a vapor chamber module for high-power LED cooling in natural convection. The size of the vapor chamber was 82.5 × 68 × 5 mm^3^, and the wick was 0.18 mm thick sintered copper powder. Compared to copper and aluminum plates, the vapor chamber reduced the thermal spreading resistance by 34% and 56%, respectively; the temperature difference was reduced by 4 °C and 6 °C, respectively. Wang et al. [6] proposed a vapor chamber-based plate for heat dissipation of 30 W high-power LED lamps, and its dimensions were 90 × 90 × 3 mm^3^. Compared to the LED aluminum-based plate, it reduced the temperature by more than 10 °C and has a maximum effective thermal conductivity of 965 W/(m·K) at 187.5 W/cm^2^. Wang et al. [7] continued to study the thermal performance of the LED vapor chamber substrate, finding that when the input power was above 5 W, the thermal performance of the vapor chamber was better than that of the copper plate. The thermal resistances of the copper plate and vapor chamber were 0.41 K/W and 0.38 K/W at 5 W, respectively.

As for the novel inner wick structure and condensation structure design for LED-cooled vapor chamber, Hsieh et al. [8] combined a rectangular aluminum groove and a copper mesh to enhance the performance of the composite structure vapor chamber of the LED lamp module. Its dimensions were 150 × 31 × 5.5 mm^3^. The results showed that, compared to a commercial substrate, the highest vapor chamber-based temperature was reduced by 28%. Ju et al. [9] incorporated composite wicks with two integrated copper powder structures: a low-thermal-resistance spreading layer and a liquid supply structure inside the vapor chamber for thermal management of high-power optoelectronic devices. They demonstrated an evaporation resistance of 0.075 (K·cm^2^)/W, whilst dissipating 1500 W from a 4 cm^2^ heating area. Wang et al. [10] fabricated a condensation surface with parallel-arranged micro-fins for an LED-cooled vapor chamber, and the meshed wick was set at the evaporation surface. Its dimensions were 100 × 100 × 6 mm^3^. The results indicated that the thermal resistance of the proposed vapor chamber was lowered by 10–15%, and the temperature uniformity at the condensation surface was the same as the traditional one. Tang et al. [11] employed parallel and orthogonal microgrooves for an LED-cooled aluminum vapor chamber, and its dimensions were 150 × 150 × 2.8 mm^3^. The results showed that the junction temperatures of the vapor chamber cooling system were always lower than those of the conventional heat sink at any current, and the total thermal resistance of the vapor chamber cooling system was 16.5% lower than that of the conventional heat sink. Chen et al. [12] proposed a vapor chamber with radial Ω-shaped multi-artery re-entrant microchannels for high-power LED thermal management. They found that the vapor chamber reduced the surface temperature of the LED module by 7–27% and decreased thermal resistance by 19–48% compared to the copper plate.

To fit the LED’s package profile, the vapor chamber adopts an adaptive exterior design for thermal management. Wang et al. [13] integrated the vapor chamber and two cylindrical heat pipes for a high anti-gravity cooling system. They designed copper powder rings and a graded pore-size wick to shorten the flow path of the working fluid and improve the capillary capacity of the wick. The results indicated that the integrated vapor chamber and heat pipes showed better anti-gravity thermal performance than a conventional heat pipe. Lu et al. [14] developed a 3D T-shaped vapor chamber by integrating a circular vapor chamber and a flattened heat pipe for LED automotive headlamp cooling. The 3D vapor chamber exhibited favorable temperature uniformity, and both-sided heating was better than one-sided heating under the same input-heat load. Jian et al. [15] also fabricated a 3D T-shaped vapor chamber for LED automotive headlamp cooling. The results indicated that the thermal resistance of the extended section accounted for the largest share of the overall thermal resistance of the 3D vapor chamber.

With the ongoing trend toward higher integration, the increasingly limited space constrains the size of the vapor chamber. This has led to the development of ultra-thin vapor chambers (UTVCs). Zhang et al. [16] proposed a 1.3 mm thick 2D T-shaped UTVC for LED automotive headlamp cooling, and a two-layered screen mesh was used as a wick. The results showed that double-sided heating could have better temperature uniformity and decrease the thermal resistance compared to single-sided heating. Shao et al. [17] employed a 0.52 mm thick UTVC with phosphor in metal for high-power illumination heat dissipation, and a three-layered screen mesh was used as a wick. The results showed that the central temperature of the UTVC was maintained below 107.0 °C at 4.92 W, while that of the reference sample was 530.18 °C.

As indicated above, the wick structure plays a key role in the vapor chamber to provide capillary force for returning the liquid working fluid. Zhou et al. [18] created a radial-gradient sintered copper powder wick for a UTVC. Tian et al. [19] proposed a sintered aluminum powder wick for a vapor chamber. Liu et al. [20] presented a multilayer composite mesh wick for a UTVC. Yang et al. developed a multilayer aluminum mesh [21] for a UTVC. Yan et al. [22] proposed a groove-mesh wick for a UTVC. In previous studies, sintered powder, screen, and groove have been widely used as wick structures in vapor chambers. As vapor chamber thickness decreases, the traditional wick structure can be eliminated, and the vapor chamber becomes wickless [23,24]. Kumar et al. [23] used an aqueous solution of a surface-active ionic liquid as a working fluid for a wickless vapor chamber. Rahimi et al. [24,25] featured a copper high-temperature conductive microporous coating over a 25 × 25 mm^2^ area on the hot surface. However, the bare metal surface cannot return the working fluid to the evaporation section. The wettability-patterned microstructure on the inner surface of the vapor chamber assists the circulation of the working fluid. Mukhopadhyay et al. [26,27] developed a uniformly superhydrophilic evaporator surface and a superhydrophilic–hydrophobic condenser surface for a wick-free vapor chamber. Luo et al. [28] used Laplace pressure differences between hydrophilic and hydrophobic wedge patterns to create fishbone and feather patterns that regulate condensate movement in a wick-free vapor chamber. The surface energy gradients generated by the hydrophilic and hydrophobic patterned microstructure can effectively return condensate to the evaporator. Although the wettable patterned microstructure is applied in wickless vapor chambers, it is rarely reported in UTVCs for LED cooling.

This paper introduces the superhydrophilic pattern microstructures (SPMS) to a 1.3 mm thick UTVC for LED lamp thermal management. The superhydrophilic pattern microstructures are produced via laser ablation. The geometry, pillar arrangement, and laser ablation parameters are distinguished from our previous work [29,30], and the details can be seen in Section 2.1. The heat transfer performance of this novel UTVC with SPMS was experimentally studied under different condensation temperatures in the horizontal orientation. This study can provide some useful design guidelines for a UTVC-based LED cooling module.

## 2. Experimental Method

### 2.1. Design and Fabrication of the Circular UTVC

**Figure 1:**
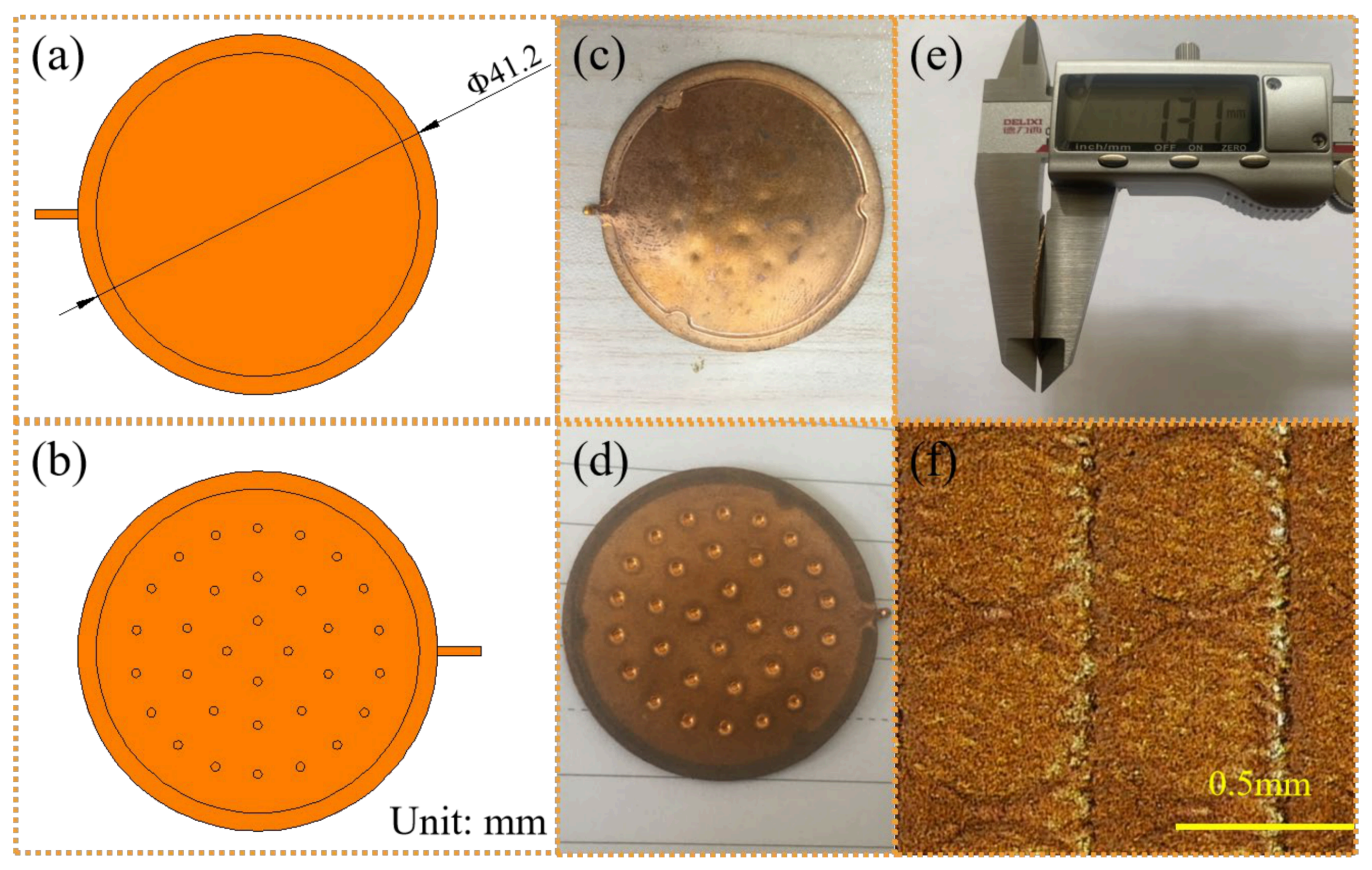
Circular UTVC: (**a**) schematic diagram of the bottom side; (**b**) schematic diagram of the upper side and the distribution of the support pillars; (**c**) photograph of the bottom side; (**d**) photograph of the upper side; (**e**) thickness of the circular UTVC; (**f**) superhydrophilic patterned microstructure inside the circular UTVC.

A circular UTVC was designed and fabricated in this study, whose schematic diagram, photograph, and inner patterned microstructure are shown in Figure 1. The circular UTVC has a diameter of 41.2 mm and a thickness of 1.3 mm, and it consists of a bottom cover, an upper cover, and a charging tube. The material of the circular UTVC is red copper (C1100). The thicknesses of the upper and bottom covers are both 0.35 mm.

**Figure 2:**
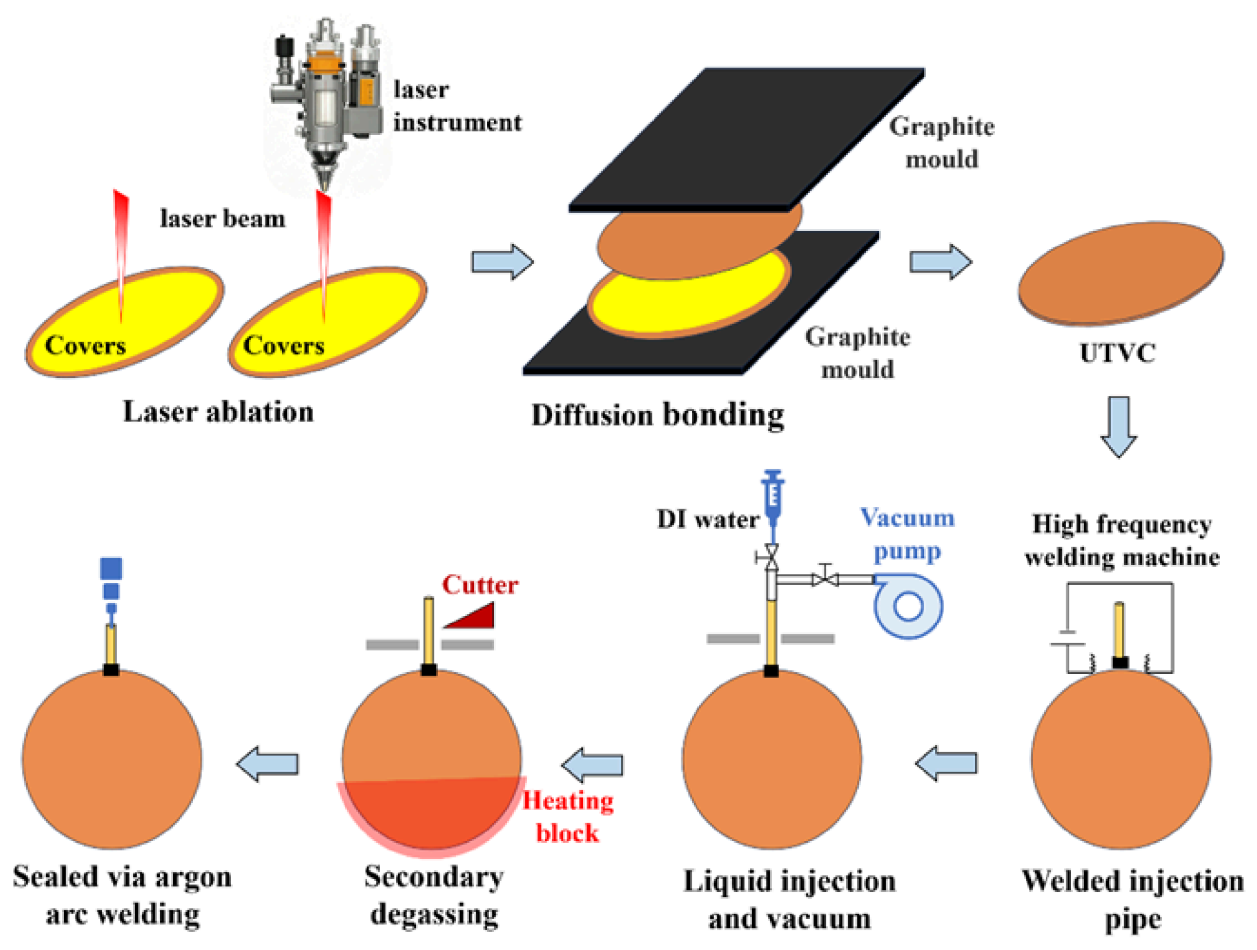
The fabrication process of the circular UTVC.

The fabrication process of the circular UTVC is shown in Figure 2. From Figure 2, the bottom cover was stamped using molds to form a chamber with an internal diameter of 37 mm and an internal height of 0.6 mm. To prevent deformation of the UTVC, 32 support pillars were included, as shown in Figure 1b. The support pillars were stamped from the upper cover and bonded at their tips to the bottom cover, thereby also providing shorter flow paths for returning condensate rapidly. Each pillar had a diameter of 1 mm and was arranged on three concentric circles: 4 pillars on a circle of diameter 7 mm, 10 pillars on a circle of diameter 17 mm, and 18 pillars on a circle of diameter 28.2 mm.

To eliminate the thickness of the wick structure and the total mass of the UTVC, the traditional wick structure was replaced by circular superhydrophilic patterned microstructure (SPMS) arrays. The circular SPMS arrays were fabricated on the upper and bottom covers by laser ablation. Laser ablation was performed at 54 W power, 2500 mm/s scanning speed, 250 ns pulse width, 40 kHz pulse repetition rate, and 2-time scanning. The depth of the SPMS was 0.026 mm. The enlarged view of the circular SPMS arrays is depicted in Figure 1f. The diameter of each circular SPMS is 0.5 mm, and the pitch of the adjacent circular SPMS is 0.6 mm. Our previous studies proved that the static contact angle of 5 μL of deionized water on the SPMS by laser ablation is near 0° [29]. The copper surface ablated by laser ablation is superhydrophilic, which has also been reported in references [26,27,28], even though their patterns are different. This superhydrophilicity indicates that the SPMS can transport water and eliminate the water bridge between the bottom and upper covers. Furthermore, the SPMS on the bottom sheet can enhance evaporative heat transfer by providing additional nucleation sites. Furthermore, the heat transfer performance of untreated bare VC and wickless VC with SPMS was evaluated in our previous study [29]. Compared with the untreated bare wickless VC, the wickless VC incorporating SPMS exhibited a 10.98% reduction in thermal resistance and an 18.75% increase in maximum heat transfer capacity. It is indicated that the laser-ablated SPMS arrays can enhance the heat transfer performance of the VC compared to the untreated bare VC. However, there is no untreated bare UTVC with Ø41.2 × 1.3 mm^2^ to compare with the proposed circular UTVC with SPMS in this study, and the benefit of the SPMS arrays is not demonstrated.

After laser ablation, the two covers were bonded together by diffusion bonding. A 1.0 mm outer diameter charging tube was welded to the UTVC by high-frequency welding. Owing to its high surface tension, high latent heat, and low viscosity [31], degassed deionized water was employed as the working fluid in the UTVC. The filling mass of the degassed deionized water was 0.12 g, and the corresponding filling ratio was 18.6% of the total volume of the container. The filling ratio is a trade-off between heat transfer performance and charging working fluid cost. In the previous study, the optimal filling ratio of UTVC with SPMS was 42.8%, but considering the reduction in the charging amount and the successful management of heat dissipation at 25 W, a filling ratio of 19% was selected, which ranked third-best in the previous literature [29]. The coarse and fine evacuation and heat-degassing process would remove the non-condensable gas in the UTVC and maintain the vacuum degree below 10 Pa. The charging tube was sealed using argon arc welding.

### 2.2. Experimental Apparatus

**Figure 3:**
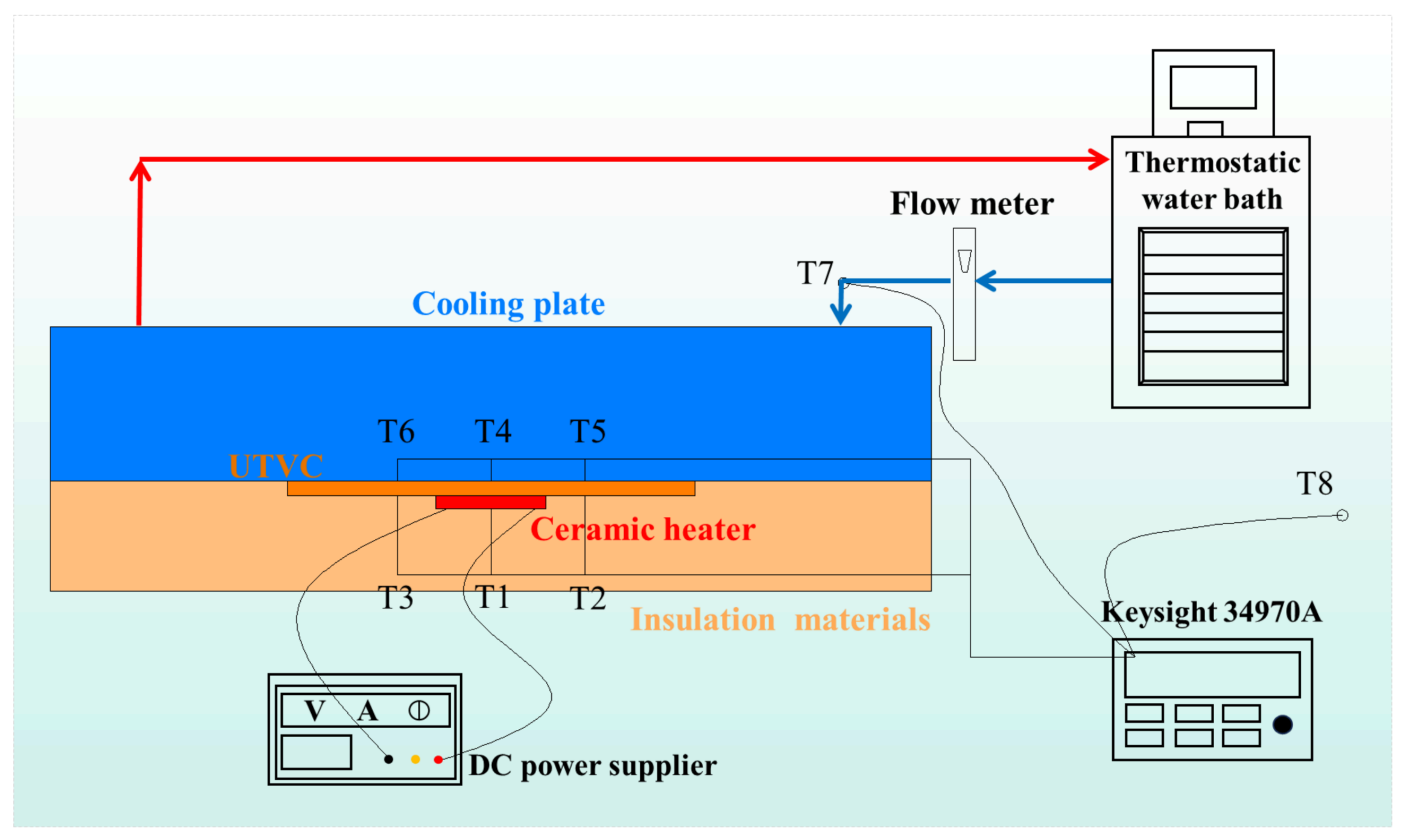
The schematic diagram of the experimental apparatus.

The schematic diagram of the experimental apparatus is shown in Figure 3. A ceramic heater was used to simulate the LED lamp for heating, placed at the center of the bottom side of the circular UTVC, with a heating area of 10 × 10 mm^2^. The ceramic heater was powered by an adjustable direct-current power supplier with a voltmeter and ammeter with accuracies of ±0.01 V and ±0.001 A (Maisheng MS-605DS, Maihao Electronic Co., Ltd., Dongguan, China). The cooling plate with a cooling area of 80 × 80 mm^2^ was in contact with the upper side of the circular UTVC. The constant-temperature cooling water was supplied by the low-temperature thermostatic water bath with a temperature control accuracy of ±0.1 °C. The cooling water flow rate was controlled by the flow meter with an accuracy of 2% at full scale.

**Figure 4:**
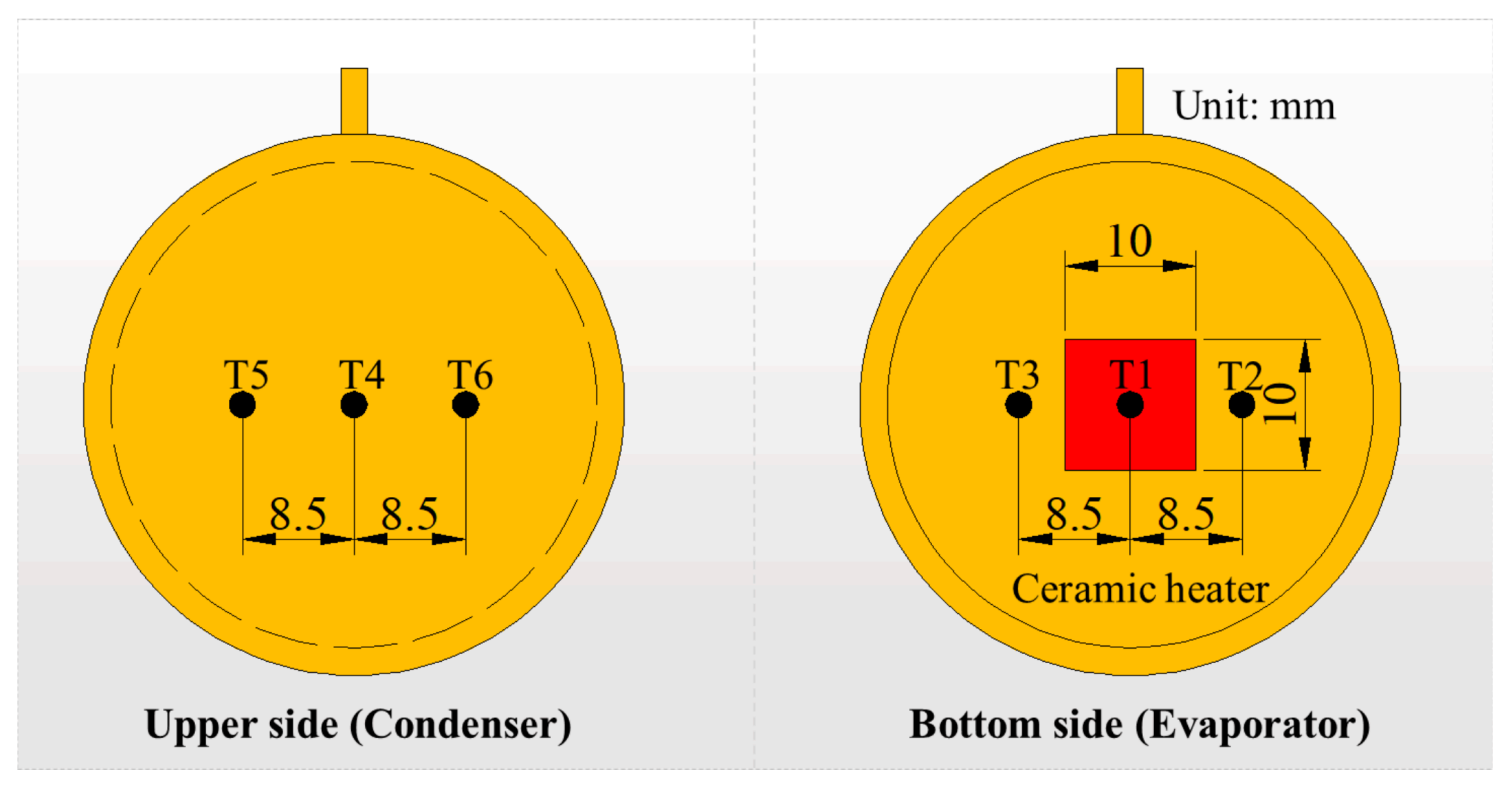
Thermocouple positions on the circular UTVC.

A Keysight 34970A data acquisition instrument with a temperature acquisition module (Keysight 34901A, Keysight Technologies, Beijing, China) and K-type thermocouples (accuracy ±0.1 °C) was used to measure and record temperatures at various positions on the circular UTVC, at the cooling plate inlet, and in the ambient air. Figure 4 shows the specific thermocouple locations. In Figure 4, thermocouple *T*1 was placed at the center of the ceramic heater; *T*2 and *T*3 were on the bottom side of the circular UTVC at a radius of 8.5 mm from the center; *T*4 was at the center of the upper side of the circular UTVC; *T*5 and *T*6 were on the upper side of the circular UTVC at a radius of 8.5 mm from the center, directly above *T*2 and *T*3, respectively; *T*7 was at the cooling plate inlet; *T*8 was in the ambient air to ensure that the ambient temperature remained stable. To reduce contact resistance, thermal grease with a thermal conductivity of 3.05 W/(m·K) was applied between the circular UTVC and the ceramic heater, and between the circular UTVC and the cooling plate. The thickness of thermal grease between the circular UTVC and the ceramic heater was 0.1 mm, and the grease between the circular UTVC and the cooling plate was 0.3 mm. The entire setup was wrapped with insulation cotton to minimize heat loss to the air.

To study the effect of condensation temperature, the cooling plate inlet temperature was set to 25 °C, 30 °C, 35 °C, and 40 °C, with a flow rate of 20 L/h in all cases. Zeng et al. [32] studied the effect of the cooling water flow rate on the heat transfer performance of the VC, and they reported that the thermal resistance was hardly affected when the cooling flow rate exceeded 10 L/h. Some studies set the cooling water flow rate to 20 L/h [33,34]. All experiments were conducted at a room temperature of 25 ± 1 °C to ensure consistency. During the experiments, the vapor chamber was placed horizontally with the evaporator at the bottom. The cooling system was started first, and the DC power supplier was adjusted to an initial heating power of 5 W. After the temperature readings stabilized, the power was increased in steps of 5 W until it exceeded 25 W, at which point the test was stopped. This ensured that temperature *T*1 would not exceed 140 °C. The test was repeated twice to confirm the reliability and accuracy of the experimental data.

### 2.3. Data Reduction and Uncertainty Analysis

The following boundary condition assumptions should be specified in the data reduction process:

(1) Heat conduction through the heater–UTVC–cooling plate stack is treated as one-dimensional;
(2) The heat flux over the 10 × 10 mm^2^ heater area is considered uniform;
(3) The contact resistance beyond the stated thermal grease conductivity is neglected.

The temperature uniformity of the UTVC can be evaluated by the condenser temperature difference, which is the maximum temperature minus the minimum temperature between all condenser thermocouples. There are three thermocouples (*T*4, *T*5, and *T*6) on the condenser. *T*4 is at the center of the condenser, where the maximum temperature occurs. *T*5 and *T*6 are equidistant from the center and located at the edge, and the smaller one represents the minimum temperature on the condenser. Therefore, the condenser temperature difference can be calculated as follows:

(1) $$\Delta T=\max\left(T_{4},T_{5},T_{6}\right)-\min\left(T_{4},T_{5},T\right)=T_{4}-\min\left(T_{5},T_{6}\right)$$

where *T*4, *T*5, and *T*6 denote the temperature locations shown in Figure 4.

The thermal resistance is used to assess the heat transfer performance of the UTVC. The heater-to-vapor-chamber thermal resistance and system thermal resistance are defined as follows:

(2) $$R_{VC}=\frac{\left(T_{1}+T_{2}+T_{3}\right)/3-\left(T_{4}+T_{5}+T_{6}\right)/3}{Q}$$

(3) $$R_{sys}=\frac{T_{1}-T_{7}}{Q}$$

where *T*1, *T*2, *T*3, and *T*7 denote the temperature locations shown in Figure 4, and *Q* denotes the input-heat load, which equals *U* × *I* (*U* denotes voltage and *I* denotes electric current). Please note that using the inlet water temperature rather than the mean or outlet water temperature biases the system thermal resistance.

The measurement uncertainty of the K-type thermocouples is ±0.1 °C, and the uncertainties of the voltmeter and ammeter are ±0.01 V and ±0.001 A, respectively.

The relative uncertainties of derived parameters such as thermal resistance and condenser temperature difference are calculated using the error propagation formula. For a function *y* = *f*(*x*_1_, *x*_2_, …, *x*_*n*_) with independent variables having uncertainties *δx*_1_, *δx*_2_, …, *δx_n_*, the error propagation is expressed as follows [35]:

(4) $$\frac{\delta y}{y}=\frac{{\left[\sum_{i=1}^{n}{\left(\frac{\partial y}{\partial x_{i}}\delta x_{i}\right)}^{2}\right]}^{\frac{1}{2}}}{y}$$

Substituting Equations (1), (2), and (3) into Equation (4), respectively, the equations for relative uncertainties of the condenser temperature difference, the heater-to-vapor-chamber thermal resistance, and system thermal resistance are obtained as follows:

(5) $$\frac{\delta \left(\Delta T\right)}{\Delta T}=\frac{\sqrt{2}\delta T}{T_{4}-\min\left(T_{5},T_{6}\right)}$$

(6) $$\frac{\delta R_{VC}}{R_{VC}}={\left\{{\left[\frac{\sqrt{6}\delta T}{\left(T_{1}+T_{2}+T_{3}\right)-\left(T_{4}+T_{5}+T_{6}\right)}\right]}^{2}+{\left(\frac{\delta U}{U}\right)}^{2}+{\left(\frac{\delta I}{I}\right)}^{2}\right\}}^{\frac{1}{2}}$$

(7) $$\frac{\delta R_{sys}}{R_{sys}}={\left[{\left(\frac{\sqrt{2}\delta T}{T_{1}-T_{7}}\right)}^{2}+{\left(\frac{\delta U}{U}\right)}^{2}+{\left(\frac{\delta I}{I}\right)}^{2}\right]}^{\frac{1}{2}}$$

Calculations show that the uncertainties of the condenser temperature difference, heater-to-vapor-chamber thermal resistance, and system thermal resistance are less than 12.03%, 20.39%, and 9.12%, respectively.

## 3. Results and Discussion

### 3.1. Temperature Distribution of the Novel UTVC

**Figure 5:**
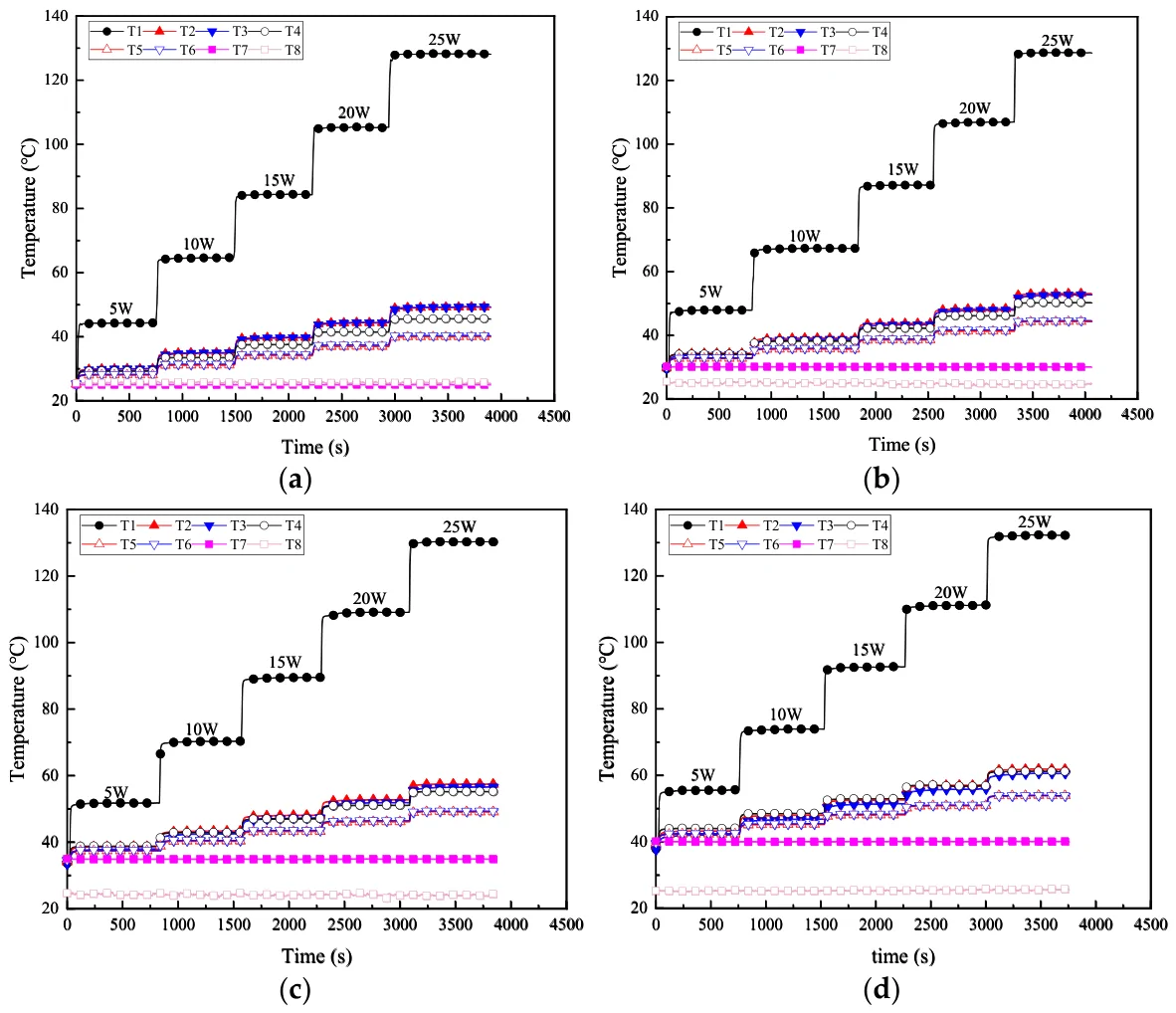
Operating temperature profile of the circular UTVC with SPMS arrays at cooling plate inlet temperatures of (**a**) 25 °C; (**b**) 30 °C; (**c**) 35 °C; (**d**) 40 °C, and input-heat load ranging from 5 to 25 W. (The *x*-axis unit is seconds, and the *y*-axis unit is °C).

To facilitate a more intuitive assessment of the UTVC’s startup performance, Figure 5 shows the real-time operating temperature profile of the circular UTVC with SPMS arrays at various measuring sites, when the cooling plate inlet temperature varies from 25 to 40 °C. The input-heat load is increased stepwise from 5 W to 25 W in 5 W increments. As shown in Figure 5, as the input-heat load increases, the ceramic heater temperature (*T*1) rises rapidly and then stabilizes for a certain period within the cooling plate inlet temperature range. Table 1 lists the *T*1 transition startup time and temperature increase rate at each power step across cooling plate inlet temperatures. The startup time is the time to 95% of the steady-state temperature. When the maximum temperature fluctuation is less than 0.3 °C, the temperature reaches a stable condition. The temperature increase rate is defined as the temperature increment during the startup time divided by the startup time. It can be seen that the startup time decreases with cooling plate inlet temperature, while the temperature increase rate increases with cooling plate inlet temperature. Since the data acquisition system collects data at a time interval of 2 s, the accuracy of the startup time is only 2 s, resulting in the same startup time for each power adjustment.

Temperatures at the evaporator (*T*2 and *T*3) and condenser (*T*4, *T*5, and *T*6) likewise increase rapidly before stabilizing for a period, but the increases are smaller than that of the ceramic heater. The slightly longer response times compared to the ceramic heater are attributed to the time delay caused by vapor diffusion. The maximum temperature fluctuation is less than 0.3 °C when the temperatures reach a stable condition. It is indicated that the circular UTVC with interior SPMS arrays achieves a state of vapor–liquid equilibrium. At all operating cooling plate inlet temperatures, the proposed circular UTVC with SPMS arrays can stably dissipate 25 W input-heat load and maintain the ceramic heater temperature below 140 °C.

Furthermore, Figure 5 shows that the trends of *T*2 and *T*3 on the evaporator with increasing input-heat load are consistent, and similarly for *T*5 and *T*6 on the condenser, indicating that vapor spreads uniformly in all directions within the UTVC.

**Figure 6:**
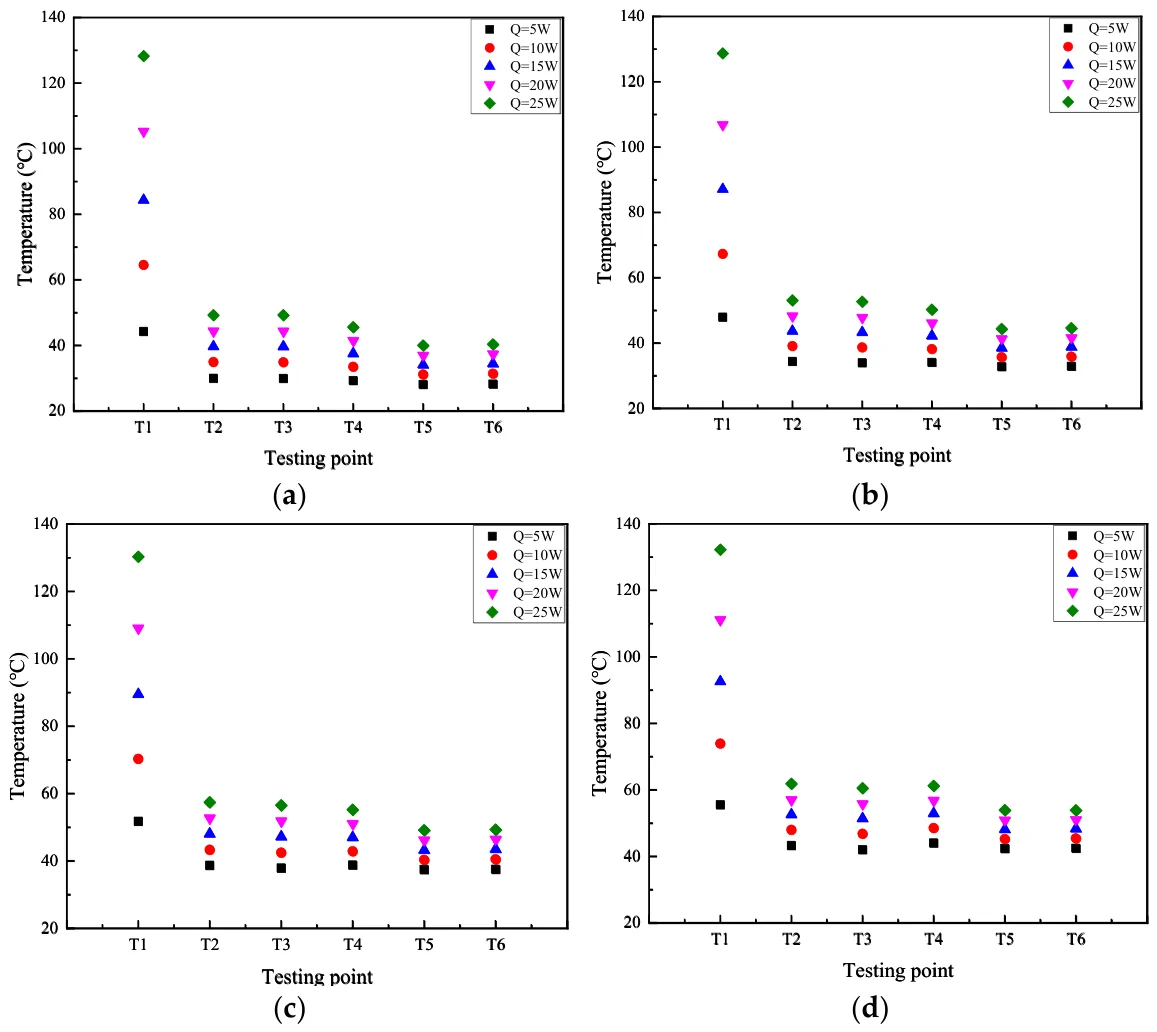
Average temperature distribution on the circular UTVC with SPMS arrays with changes in the input-heat load at cooling plate inlet temperatures of (**a**) 25 °C; (**b**) 30 °C; (**c**) 35 °C; (**d**) 40 °C, and input-heat load ranging from 5 to 25 W. (No unit is given for the *x*-axis, and the *y*-axis unit is °C).

Figure 6 presents the average temperatures at each measuring point on the circular UTVC with SPMS arrays (*T*1–*T*6) under steady-state conditions at each power level when the cooling plate inlet temperatures vary from 25 to 40 °C. It can be seen from Figure 6 that the evaporator temperatures (*T*1–*T*3) are higher than those on the condenser (*T*4–*T*6) when the cooling plate inlet temperatures range from 25 to 35 °C. When the cooling plate inlet temperature is 40 °C, the center temperature *T*4 is slightly larger than the temperatures *T*2 and *T*3 on the evaporator. This is because the lower the cooling plate inlet temperature, the faster the heat dissipation rate at the condenser. Furthermore, the center temperatures on the evaporator (*T*1) and condenser (*T*4) are higher than the both-side temperatures (8.5 mm from the center) on the evaporator (*T*2–*T*3) and condenser (*T*5–*T*6). As the cooling plate inlet temperature increases, the heat dissipation rate at the condenser decreases, which results in a higher temperature of *T*4 than that of *T*2 and *T*3 at a higher cooling plate inlet temperature of 40 °C.

From Figure 6, it is also evident that as input-heat load increases, the temperature differences between the ceramic heater and the UTVC measuring points grow. The ceramic heater temperature *T*1 is slightly higher than the temperatures on the UTVC at low-input-heat load. At high-input-heat load, the ceramic heater temperature *T*1 is much higher than the temperatures on the UTVC. The temperature differences between *T*1 and the average temperature *T*2–*T*3 are listed in Table 2. At 5 W, the temperature differences between *T*1 and the average temperature *T*2–*T*3 are 14.32, 13.75, 13.46, and 12.88 °C, respectively, at cooling plate inlet temperatures of 25, 30, 35, and 40 °C. At 20 W, the temperature differences between *T*1 and the average temperature *T*2–*T*3 are 60.97, 58.80, 56.83, and 54.74 °C, respectively, at cooling plate inlet temperatures of 25, 30, 35, and 40 °C. This is because the thickness of the SPMS arrays is small, which cannot provide additional capillary channels to rapidly backflow the condensate. Moreover, the heating area is small. The ceramic heater temperature *T*1 rises more significantly at high input-heat load. Nevertheless, the temperature differences among the UTVC measuring points themselves do not exceed 10 °C, suggesting that the circular UTVC with SPMS arrays has a favorable temperature uniformity.

**Figure 7:**
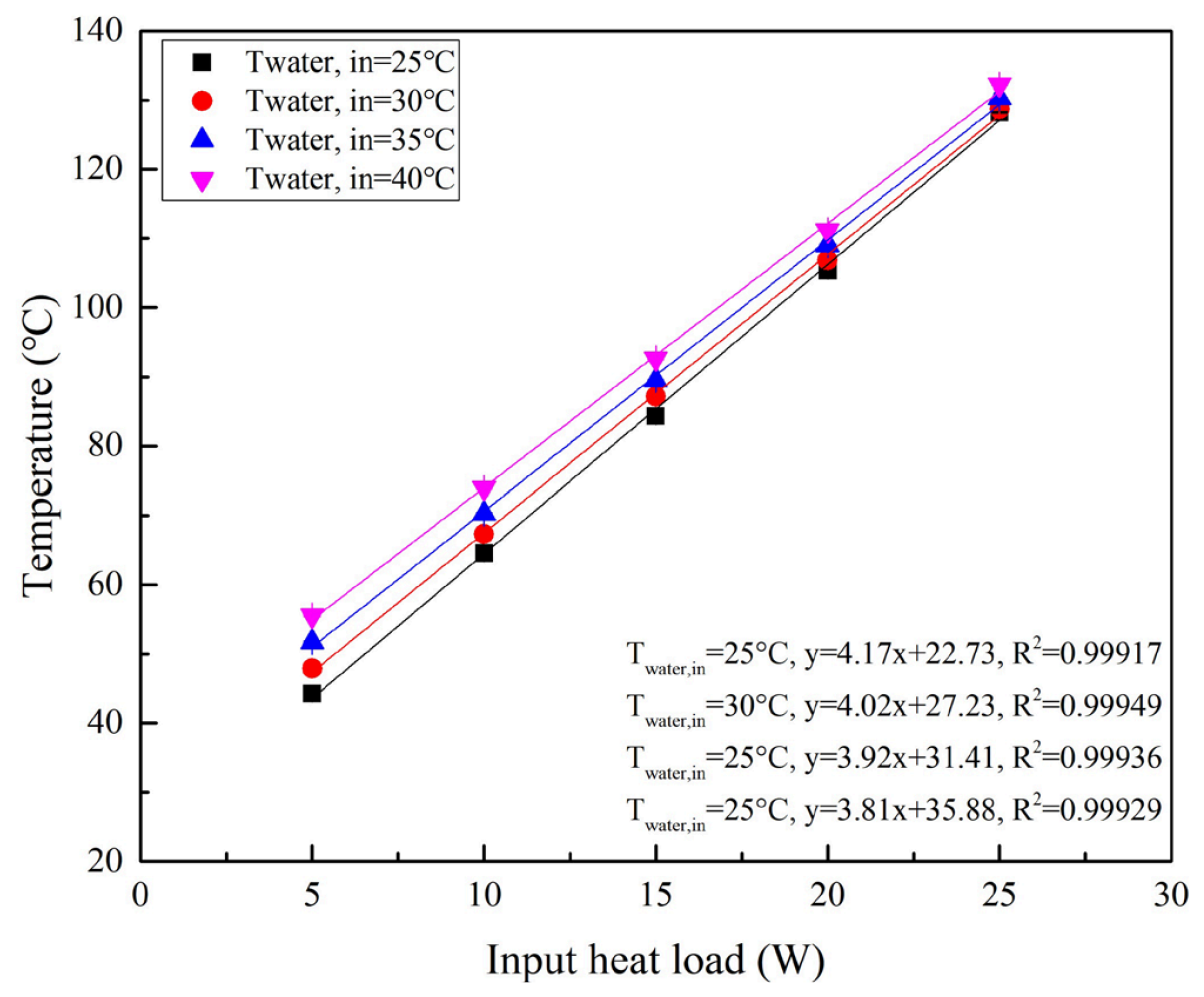
The temperature *T*1 varies with the input-heat load at cooling plate inlet temperatures ranging from 25 to 40 °C, and input-heat load ranging from 5 to 25 W. (The *x*-axis unit is W, and the *y*-axis unit is °C).

Figure 7 compares the ceramic heater temperature *T*1 changes with input-heat load under different cooling plate inlet temperatures. Figure 7 shows that as the cooling plate inlet temperature increases, the ceramic heater temperature *T*1 rises, but the ceramic heater temperature increase is smaller than that of the cooling water. As the input-heat load increases, the temperature differences at different cooling plate inlet temperatures become smaller. At 25 W, the temperatures are 128.24, 128.70, 130.27, and 132.23 °C for cooling plate inlet temperatures of 25, 30, 35, and 40 °C, respectively. This can be attributed to partial dry-out (where the working fluid fails to fully wet the heated area), and less condensate backflow to the evaporator at high-input-heat load. Moreover, the regression fits between temperature *T*1 and input-heat load are obtained at cooling plate inlet temperatures ranging from 25 to 40 °C. All R^2^ values are larger than 0.999, demonstrating good linearity between temperature *T*1 and input-heat load.

### 3.2. Temperature Uniformity on the Condenser

**Figure 8:**
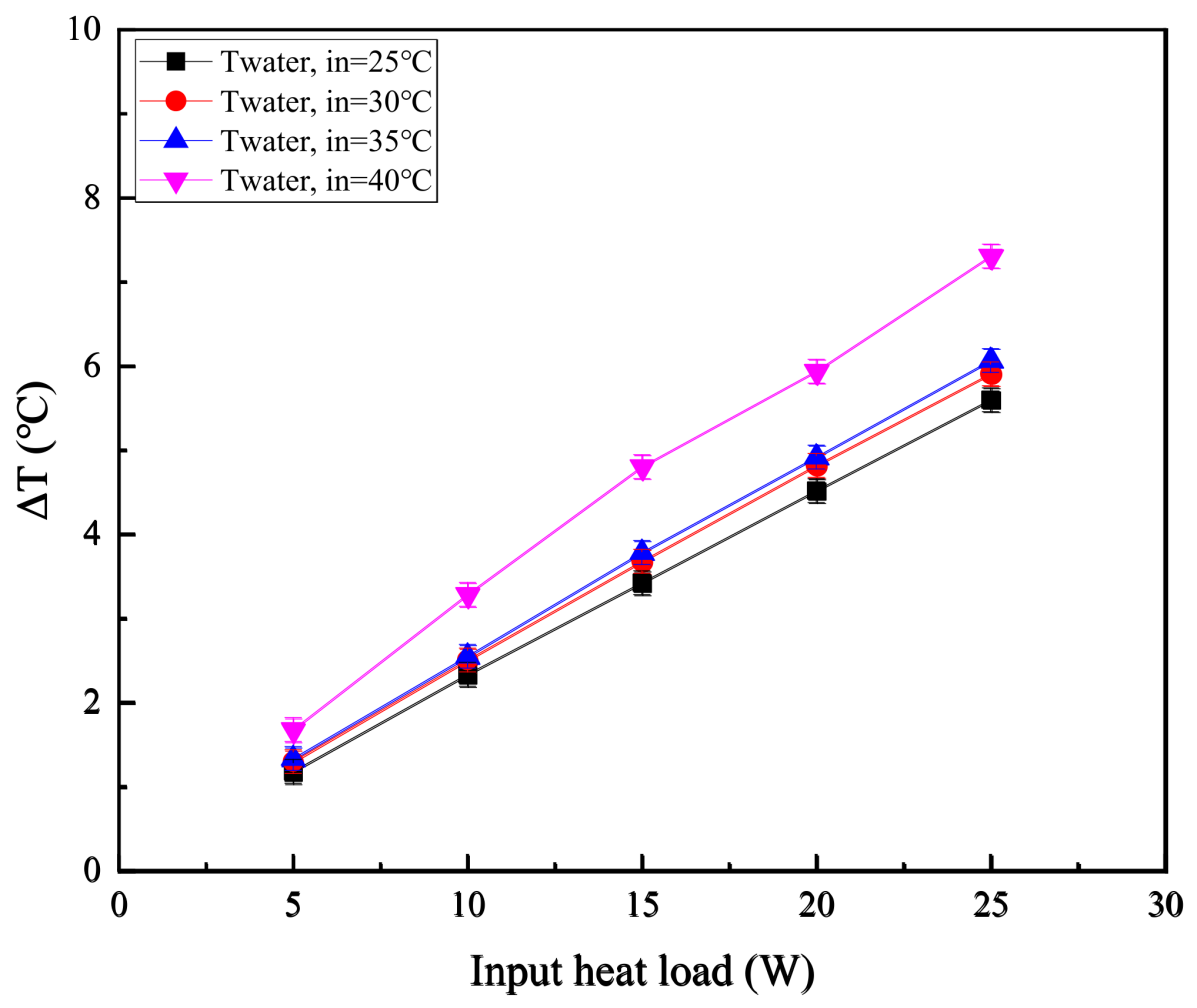
Condenser temperature difference at cooling plate inlet temperatures ranging from 25 to 40 °C, and input-heat load ranging from 5 to 25 W. (The *x*-axis unit is W, and the *y*-axis unit is °C).

Figure 8 shows the condenser temperature differences as a function of input-heat load for different cooling water temperatures. As seen in Figure 8, the condenser temperature difference increases with input-heat load, indicating poorer temperature uniformity at high-input-heat load. This is attributed to an increase in vapor diffusion resistance at higher heat fluxes. Figure 8 also shows that at the same input-heat load, higher cooling water temperatures lead to larger condenser temperature differences. The maximum value of 7.3 °C occurs at 40 °C cooling plate inlet temperature and 25 W input-heat load. Additionally, at the same power level, the curves for 25 °C, 30 °C, and 35 °C cooling plate inlet temperatures are close to each other. However, at 40 °C, the condenser temperature difference is significantly larger than that at 35 °C, suggesting that the heat transfer performance begins to deteriorate when the cooling water temperature rises to 40 °C.

### 3.3. Thermal Resistance Analysis

**Figure 9:**
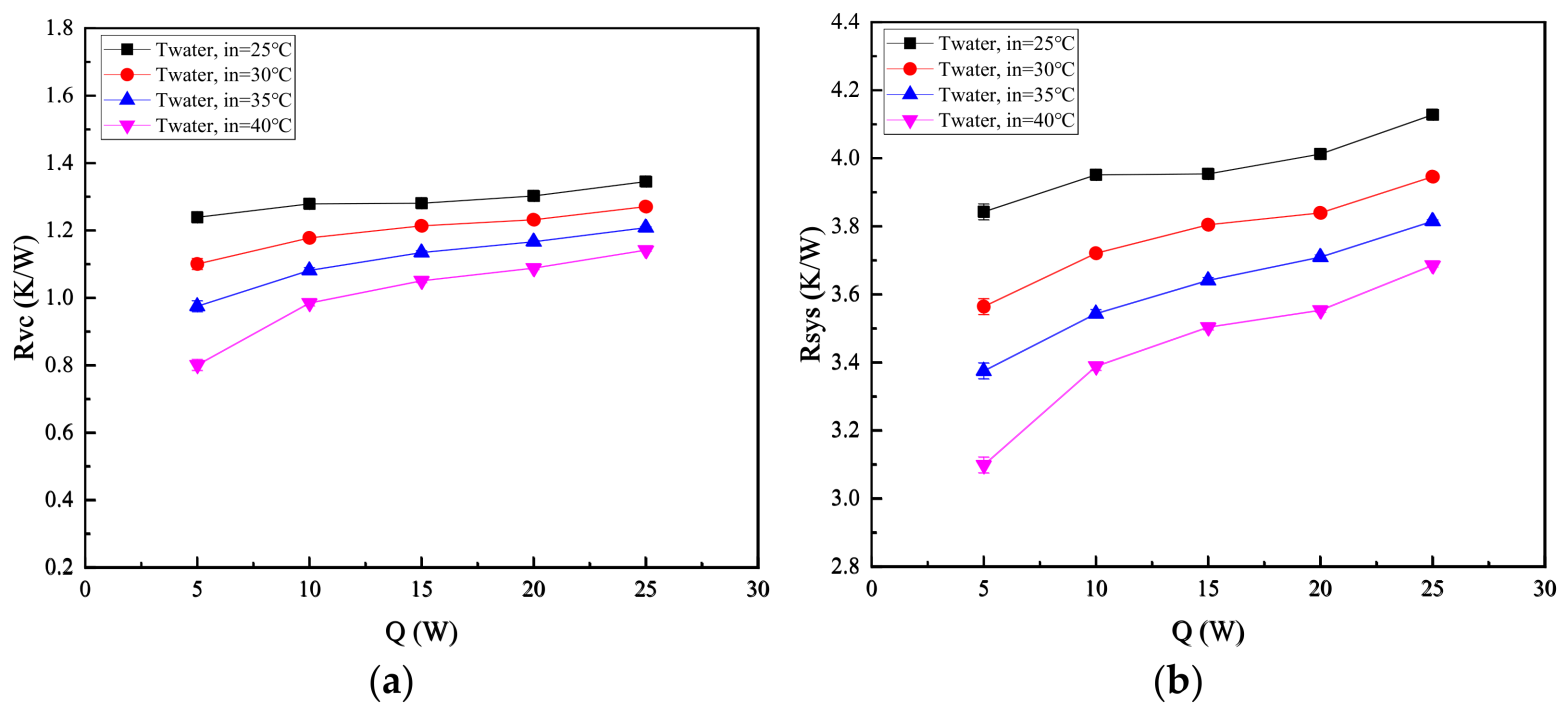
Thermal resistance at cooling plate inlet temperatures ranging from 25 to 40 °C, and input-heat load ranging from 5 to 25 W: (**a**) heater-to-vapor-chamber thermal resistance; (**b**) system thermal resistance. (The *x*-axis unit is W, and the *y*-axis unit is K/W).

Figure 9 presents the heater-to-vapor-chamber thermal resistance and the system thermal resistance as functions of input-heat load for different cooling plate inlet temperatures. Note that the thermal resistance of the heater and thermal grease is 0.192 K/W based on the one-dimensional thermal resistance estimation. The thermal resistance of the heater and thermal grease remains constant in all operating conditions due to the constant thermal conductivity of the heater and thermal grease. Figure 9 shows that at the same cooling plate inlet temperature, both the heater-to-vapor-chamber thermal resistance and the system thermal resistance increase slightly with heating power, implying an increase in vapor resistance and the onset of partial dry-out (where the working fluid fails to fully wet the heated area) near the center of the evaporator [36,37] at higher heat fluxes. This can be attributed to the thin thickness of the SPMS arrays, which cannot offer adequate liquid channels for the condensate flowing back to the evaporator.

Figure 9 also indicates that at the same heating power, increasing the cooling plate inlet temperature led to a gradual decrease in thermal resistance. This is because higher condensation temperatures reduce the friction coefficients of both vapor and liquid, and decrease the heat flux of the onset of nucleate boiling, thereby lowering the liquid return resistance. The minimum values of both the heater-to-vapor-chamber thermal resistance and system thermal resistance occur at a cooling plate inlet temperature of 40 °C and an input-heat load of 5 W, with values of 0.80 K/W and 3.10 K/W, respectively.

### 3.4. Comparison with Other Studies

The thermal performance comparisons of the circular UTVC with SPMS in this work and the other VCs in references in horizontal state are listed in Table 3. The equivalent thermal resistance and heat flux are, respectively, given by the following equations:

(8) $$R_{eq}=R\cdot A_{hs}$$

(9) $$q=\frac{Q}{A_{hs}}$$

where *A_hs_* represents the heating area. The circular UTVC with SPMS shows better equivalent thermal resistance and heat flux than those of vapor chambers in references [15,16,29], even though the equivalent thermal resistance of the circular UTVC with SPMS includes the thermal resistance of the heater and thermal grease. By enlarging the condenser surface (including the filling mass), increasing thickness, and using a superhydrophilic–hydrophobic condenser surface, the equivalent thermal resistance will be decreased, and the *q*_max_ will be increased [24,26].

## 4. Conclusions

This paper proposed a novel 1.3 mm thick circular UTVC with SPMS arrays for LED lamp cooling. SPMS arrays have been successfully created on the upper and bottom covers. This eliminates the traditional wick structure, which can provide lateral dispersal via surface energy gradients to return the condensate. The heat transfer performance was experimentally analyzed under different cooling water temperatures in the horizontal orientation. The main conclusions are as follows:

(1) As the input-heat load increases, temperatures at the proposed UTVC with SPMS arrays rise rapidly and then stabilize after a certain period, but the increases are much smaller than that of the ceramic heater. Nevertheless, the temperature differences among the UTVC measuring points do not exceed 10 °C. This indicates that the proposed UTVC achieves a state of vapor–liquid equilibrium. Furthermore, it can effectively dissipate a 25 W input-heat load and maintain the ceramic heater temperature below 140 °C.
(2) The condenser temperature difference increases with both input-heat load and cooling water temperature. The largest condenser temperature difference is 7.3 °C, which occurs at a cooling water temperature of 40 °C and an input-heat load of 25 W.
(3) Both the heater-to-vapor-chamber thermal resistance and the system thermal resistance increase with higher input-heat load and lower cooling water temperature. The minimum heater-to-vapor-chamber thermal resistance and system thermal resistance are 0.80 K/W and 3.10 K/W, respectively, and are achieved at a cooling water temperature of 40 °C and an input-heat load of 5 W.

However, the present study has some limitations: only a single sample was tested, and no reference sample with the same geometry was available; the thermal resistance included that of the heater, and a simulated LED source was used; SPMS survival after bonding and the two-phase flow circulation in the UTVC are unclear. In future studies, the proposed circular UTVC with SPMS arrays can be integrated with an LED module and electronics for real-world application. The effect of cooling water flow rate and orientation on the heat transfer performance of the proposed UTVC can be further investigated. The two-phase flow circulation in the UTVC can be visualized to understand the heat transfer mechanism of the novel UTVC, and a two-dimensional heat spreading model can be established to confirm the measured thermal resistance. A novel cooling method, such as an integrated thermoelectric generator and heat sink [38] with UTVC, can be introduced for LED lamp thermal management.

## Figures and Tables

**Table 1:** The transition startup time and temperature increase over time.

Power Step	Cooling Temperature = 25 °C		Cooling Temperature = 30 °C		Cooling Temperature = 35 °C		Cooling Temperature = 40 °C	
	Startup Time (±2 s)/s	Temperature Increase Rate/(°C/s)	Startup Time (±2 s)/s	Temperature Increase Rate/(°C/s)	Startup Time (±2 s)/s	Temperature Increase Rate/(°C/s)	Startup Time (±2 s)/s	Temperature Increase Rate/(°C/s)
0 to 5 W	20	0.95	14	1.31	12	1.50	10	1.76
5 to 10 W	20	1.01	14	1.38	12	1.54	10	1.84
10 to 15 W	20	0.99	14	1.42	12	1.60	10	1.87
15 to 20 W	20	1.05	14	1.41	12	1.63	10	1.85
20 to 25 W	20	1.15	14	1.56	12	1.77	10	2.10

**Table 2:** Temperature differences between *T*1 and the average temperature *T*2–*T*3.

Input-Heat Load/W	Cooling Temperature = 25 °C	Cooling Temperature = 30 °C	Cooling Temperature = 35 °C	Cooling Temperature = 40 °C
	Temperature Difference/°C	Temperature Difference/°C	Temperature Difference/°C	Temperature Difference/°C
5	14.32	13.75	13.46	12.88
10	29.65	28.41	27.41	26.53
15	44.69	43.62	41.85	40.62
20	60.97	58.80	56.83	54.74
25	79.01	75.84	73.32	71.06

**Table 3:** The thermal performance in horizontal state comparisons of other studies.

Information of VCs in References	Thermal Performance
This study: UTVC, Ø41.2 × 1.3 mm^2^, superhydrophilic patterned microstructure, water, FR of chamber volume = 18.6%	*R*_eq,VC,min_ = 0.80 (K·cm^2^)/W, *R*_eq_,_sys,min_ = 3.10 (K·cm^2^)/W, *q*_max_ = 25 W/cm^2^
Ref. [15]: 3D T-shape VC, the circular vapor chamber Ø39.5 mm and H 4 mm, the flattened heat pipe 57.3 × 12.5 × 2.5 mm^3^, copper powder, water, FR not mentioned	Double-side heating: *R*_eq,min_ = 1.504 (K·cm^2^)/W, *q*_max_ = 4.224 W/cm^2^
Ref. [16]: 2D T-shape UTVC, horizontal stripe 30 × 13 × 1.3 mm^3^, vertical stripe 46.5 × 13 × 1.3 mm^3^, screen mesh, water, FR of the wick = 100%	One-side heating: *R*_eq,min_ = 1.803 (K·cm^2^)/W, *q*_max_ = 15 W/cm^2^; Double-side heating: *R*_eq,min_ = 2.874 (K·cm^2^)/W, *q*_max_ = 7.5 W/cm^2^
Ref. [23]: VC, 40 × 40 × 3.5 mm^3^, wickless, aqueous solution of surface-active ionic liquid, FR of chamber volume = 100%	*R*_eq,min_ = 0.85 (K·cm^2^)/W, *q*_max_ = 120 W/cm^2^
Ref. [24]: UTVC, 80 × 80 × 1.6 mm^3^, wickless, a copper microporous coating on the hot surface, water, FR not mentioned	*R*_eq,min_ = 0.135 (K·cm^2^)/W, *q*_max_ = 200 W/cm^2^
Ref. [26]: VC, 114.3 × 63.5 × 5 mm^3^, wickless, a uniformly superhydrophilic evaporator surface and a superhydrophilic–hydrophobic condenser surface, water, FR not mentioned	*R*_eq,min_ = 0.143 (K·cm^2^)/W, *q*_max_ = 45 W/cm^2^
Ref. [29] UTVC, 70 × 70 × 1.5 mm^3^, wickless, wettable patterned microstructure, water, FR of chamber volume = 42.8%	*R*_eq,min_ = 1.314 (K·cm^2^)/W, *q*_max_ = 21.1 W/cm^2^

## Data Availability

The original contributions presented in this study are included in the article. Further inquiries can be directed to the corresponding author(s).

## Abbreviations

The following abbreviations are used in this manuscript:

LED	Light-emitting diode
SPMS	Superhydrophilic patterned microstructure
UTVC	Ultra-thin vapor chamber
VC	Vapor chamber
*I*	Electric current, A
*q*	Heat flux, W/cm^2^
*Q*	Input-heat load, W
*R_eq_*	Equivalent thermal resistance, (K·cm^2^)/W
*R_VC_*	Heater-to-vapor-chamber thermal resistance, K/W
*R_sys_*	System thermal resistance, K/W
*T*	Temperature, °C
*x*	Independent variable
*y*	Function

## References

[1] Luo X., Hu R., Liu S., Wang K. (2016). Heat and Fluid Flow in High-Power LED Packaging and Applications. Prog. Energy Combust. Sci..

[2] Ding X., Tang Y., Li Z., Chen Q., Li Y., He Y. (2015). Thermal and Optical Investigations of High Power LEDs with Metal Embedded Printed Circuit Boards. Int. Commun. Heat Mass Transf..

[3] Zhao X.J., Cai Y.X., Wang J., Li X.H., Zhang C. (2015). Thermal Model Design and Analysis of the High-Power LED Automotive Headlight Cooling Device. Appl. Therm. Eng..

[4] Arik M., Petroski J., Weaver S. Thermal Challenges in the Future Generation Solid State Lighting Applications: Light Emitting Diodes. Proceedings of the IEEE 8th ITherm Conference.

[5] Huang H.S., Chiang Y.C., Huang C.K., Chen S.L. (2009). Experimental Investigation of Vapor Chamber Module Applied to High-Power Light-Emitting Diodes. Exp. Heat Transf..

[6] Wang J.C., Wang R.T., Chang T.L., Hwang D.S. (2010). Development of 30 Watt High-Power LEDs Vapor Chamber-Based Plate. Int. J. Heat Mass Transf..

[7] Wang J.C. (2011). Thermal Investigations on LED Vapor Chamber-Based Plates. Int. Commun. Heat Mass Transf..

[8] Hsieh J.C., Huang H.J., Shen S.C. (2012). Experimental Study of Microrectangular Groove Structure Covered with Multi Mesh Layers on Performance of Flat Plate Heat Pipe for LED Lighting Module. Microelectron. Reliab..

[9] Ju Y.S., Kaviany M., Nam Y., Sharratt S., Hwang G.S., Catton I., Fleming E., Dussinger P. (2013). Planar Vapor Chamber with Hybrid Evaporator Wicks for the Thermal Management of High-Heat-Flux and High-Power Optoelectronic Devices. Int. J. Heat Mass Transf..

[10] Wang Y., Cen J., Jiang F., Cao W. (2017). Heat Dissipation of High-Power Light Emitting Diode Chip on Board by a Novel Flat Plate Heat Pipe. Appl. Therm. Eng..

[11] Tang Y., Lin L., Zhang S., Zeng J., Tang K., Chen G., Yuan W. (2017). Thermal Management of High-Power LEDs Based on Integrated Heat Sink with Vapor Chamber. Energy Convers. Manag..

[12] Chen L., Deng D., Huang Q., Xu X., Xie Y. (2020). Development and Thermal Performance of a Vapor Chamber with Multi-Artery Reentrant Microchannels for High-Power LED. Appl. Therm. Eng..

[13] Wang H., Bai P., Zhou H., Coehoorn R., Li N., Liao H., Zhou G. (2019). An Integrated Heat Pipe Coupling the Vapor Chamber and Two Cylindrical Heat Pipes with High Anti-Gravity Thermal Performance. Appl. Therm. Eng..

[14] Lu Z., Bai P., Huang B., Henzen A., Coehoorn R., Liao H., Zhou G. (2019). Experimental Investigation on the Thermal Performance of Three-Dimensional Vapor Chamber for LED Automotive Headlamps. Appl. Therm. Eng..

[15] Jian Q., Li C., Wang L. (2019). Analysis on Thermal and Hydraulic Performance of a T-Shaped Vapor Chamber Designed for Motorcycle LED Lights. Therm. Sci..

[16] Zhang Y., Liu T., Bai Y., Wang S., Zhang Q., Kang H. (2025). Thermal Performance of T-Shaped Ultra-Thin Vapor Chamber with Double-Sided Heating for LED Automotive Headlamp Cooling. Micromachines.

[17] Shao C., Yan C., Liu X., He Y., Tang Y., Zhang S. (2025). Enhancing Heat Dissipation for Laser Illumination by Utilizing the Phosphor in Metal Combined with Vapor Chamber (PiMCVC). Opt. Laser Technol..

[18] Zhou F., Zhou G., Zhou J., Jia X., Huai X. (2023). Performance Comparative Evaluation of Three Thin Vapor Chambers with Different Wick Structures. Appl. Therm. Eng..

[19] Tian X., Zheng F., Wang L., Hu Q., Liang X., Xu Y., Qin J., Zhang H., Miao J. (2025). Heat Transfer Performance of a Novel Flat Plate Heat Pipe with Sintered Aluminum Powder Wicks. Appl. Therm. Eng..

[20] Liu T., Yan W., Yang X., Wang S. (2022). Improving the Thermal Performance of Thin Vapor Chamber by Optimizing Screen Mesh Wick Structure. Therm. Sci. Eng. Prog..

[21] Yang Y., Wei Y., Yao Y., Xie Y., Yang S., Li Y., Lu L. (2025). Design and Fabrication of Ultrathin Aluminum Vapor Chambers with Layered Heterogeneous Vapor-Liquid Channel Structures. Int. J. Heat Mass Transf..

[22] Yan W., He X., Wang S., Chen K. (2025). Heat Transfer Performance of Ultra-Thin Vapor Chambers with Composite Wick for Electronic Thermal Management. Therm. Sci. Eng. Prog..

[23] Kumar N., Sinha K.N.R., Raza M.Q., Verma A., Raj R. (2020). Design, Fabrication, and Performance Evaluation of a Novel Orientation Independent and Wickless Heat Spreader. Int. J. Heat Mass Transf..

[24] Rahimi H., Lim H., Fadda D., Lee J., You S.M. (2026). Thermal Performance of Liquid-Filled TGPs: Structural and Degassing Effects. Appl. Therm. Eng..

[25] Rahimi H., Fadda D., Lee J., You S.M. (2025). Working Principles of Wickless Thermal Ground Planes. Appl. Therm. Eng..

[26] Mukhopadhyay A., Pal A., Bao C., Gukeh M.J., Mazumder S.K., Megaridis C.M. Evaluation of Thermal Performance of a Wick-Free Vapor Chamber in Power Electronics Cooling. Proceedings of the 22nd IEEE Intersociety Conference on Thermal and Thermomechanical Phenomena in Electronic Systems (ITherm).

[27] Mukhopadhyay A., Pal A., Gukeh M.J., Megaridis C.M. Thermal Performance of a Liquid-Cooling Assisted Thin Wickless Vapor Chamber. Proceedings of the 22nd IEEE Intersociety Conference on Thermal and Thermomechanical Phenomena in Electronic Systems (ITherm).

[28] Luo Q., Zhang Z., Wang C., Wu T., Cen Z. (2026). Study on Heat Transfer Performance of Wick-Free Vapor Chamber with Superhydrophilic and Superhydrophobic Self-Transport Pattern. Int. J. Therm. Sci..

[29] Liu T., Yan W., Yang X., Wang S. (2022). Thermal Performance of Wickless and Orientation Independent Thin Vapor Chambers with Wettability Patterned Micro Structure. Therm. Sci..

[30] Liu T., Zhang Y., Wang S., Zhang Q., Kang H. (2026). Experimental Research on the Heat Transfer Performance of Ultra-Thin Vapor Chambers with Composite Wicks for Electronics Cooling. Int. J. Heat Fluid Flow.

[31] Patankar G., Weibel J.A., Garimella S.V. (2020). On the Transient Thermal Response of Thin Vapor Chamber Heat Spreaders: Optimized Design and Fluid Selection. Int. J. Heat Mass Transf..

[32] Zeng J., Zhang S., Chen G., Lin L., Sun Y., Chuai L., Yuan W. (2018). Experimental Investigation on Thermal Performance of Aluminum Vapor Chamber Using Micro-Grooved Wick with Reentrant Cavity Array. Appl. Therm. Eng..

[33] Yu J., Xin Z., Zhang R., Chen Z., Li Y., Zhou W. (2022). Effect of Spiral Woven Mesh Liquid Pumping Action on the Heat Transfer Performance of Ultrathin Vapour Chamber. Int. J. Therm. Sci..

[34] Huang G., Liu W., Luo Y., Li Y., Chen H. (2021). A New Ultra-Thin Vapor Chamber with Composite Wick for Thin Electronic Products. Int. J. Therm. Sci..

[35] Moffat R.J. (1988). Describing the Uncertainties in Experimental Results. Exp. Therm. Fluid Sci..

[36] Tang Y., Yuan D., Lu L., Wang Z. (2013). A Multi-Artery Vapor Chamber and Its Performance. Appl. Therm. Eng..

[37] Huang G., Liu W., Luo Y., Li Y., Chen H. (2019). Fabrication and Thermal Performance of Mesh-Type Ultra-Thin Vapor Chambers. Appl. Therm. Eng..

[38] Hachim D.M., Al-Manea A., Al-Rbaihat R., Abed Q.A., Sadiq M., Homod R.Z., Alahmer A. (2025). Enhancing the Performance of Photovoltaic Solar Cells Using a Hybrid Cooling Technique of Thermoelectric Generator and Heat Sink. J. Sol. Energy Eng..

